# Thymidylate synthase gene (*TYMS*) polymorphisms in sporadic and hereditary breast cancer

**DOI:** 10.1186/1756-0500-5-676

**Published:** 2012-12-06

**Authors:** José da Silva Nogueira Junior, Fernando Augusto de Lima Marson, Carmen Sílvia Bertuzzo

**Affiliations:** 1 Department of Genetics, Faculty of Medical Sciences, University of Campinas, P.O. Box: 6111, Campinas, SP 13081-970, Brazil; 2 Department of Pediatrics, School of Medical Sciences, University of Campinas, P.O. Box: 6111, Campinas, SP 13081-970, Brazil; 3 Pediatrics Department, Medical Genetics Department, University of Campinas – UNICAMP, Campinas, Brazil

**Keywords:** Thymidylate synthasel, Gene polymorphisms, Breast cancer, Sporadic breast cancer, Sporadic, Hereditary breast cancer

## Abstract

**Background:**

Breast cancer (BC) is a genetic disorder characterized by growth and proliferation of breast cells in a disorderly. In Brazil, there are approximately 49.240 new cases of BC, every year. The BC etiology is still poorly understood. The BC can be sporadic (SBC) or hereditary (HBC). Recent studies have correlated gene polymorphisms with the BC, such as alterations in *thymidylate synthase gene* (TYMS), which are used to improve diagnosis and prevention of the disease. Polymorphisms in the *TYMS* gene 5’-UTR region, usually present reps double (2R) and/or triple (3R). Studies have shown that homozygous 3R/3R is overexpressed compared with 2R/2R genotype, and these polymorphic variations may contribute to individual susceptibility to the development of BC. In this context, the objective of this study was to evaluate the frequency of the *TYMS* 2R and 3R polymorphisms, comparing genotypic and allelic distribution with SBC and HBC patients.

**Methods:**

In this study we included a total of 204 subjects, 70 with BC (33 with SBC, and 37 with HBC) and 134 healthy subjects (controls). The Polymerase Chain Reaction was the method used.

**Results:**

Results demonstrated a high frequency of the 3R allele at BC, SBC, and HBC groups. The frequency of genotype 2R/3R was significantly higher in BC group. This work showed association between the 2R/3R variants (OR = 4.14, CI95% = 1.77-9.71) in the development of SBC, and 2R/2R (OR = 0.233, CI95% = 1.63-7.65) and 2R/3R (OR = 3.53, CI95% = 0.06-0.81) for developing HBC. To BC, there was association with the genotype 2R/3R (OR: 3.79, CI95% = 2.03-7.08).

**Conclusion:**

Our results show relation to the development of BC in association with the analyzed polymorphisms.

## Background

Breast cancer (BC) is a genetic disease characterized by an out of control growing breast cells, resulting in cellular proliferation, invasion of surrounding tissues and other organs, with possibility of metastasis [1]. BC is the second leading cancer in the population, it is the most common cancer among women, and the second leading cause of death within them, with approximately 460.000 deaths/year worldwide [1,2].

In recent years risk factors for BC have been identified, although the etiology of the disease is still not understood. Risk factors that contribute to the development of BC include age, ethnicity, reproduction, some kind of hormones, lifestyle, bone density, genetic factors [3] and family history [4]. The majority of hereditary breast cancer (HBC) susceptibility can be attributed to germline mutations of to Breast Cancer 1 and Breast Cancer 2 genes (*BRCA1* and *BRCA2*), which are responsible for 30-40% of HBC. Clinically, the basis of HBC is established at an early age, family history, bilateral BC, male BC, ovarian and/or tube cancer, and lower survival when compared to the sporadic form [5].

Most of BC are sporadic (SBC), resulting from gene mutations, uncorrected, located in somatic cells, and unrelated to germline mutation. Risk factors for SBC are often hormonal [6], although, it may be related to other risk factors like smoking, ionizing radiation and genotoxics agents [7].

Recently, the association of polymorphisms in some genes has been demonstrated as a possible etiologic factor of neoplasia and response to chemotherapy [8]. Molecular markers related to BC have been described as risk factors, being studied as a biomarker of diagnosis, prognosis and prevention [3,9]. Among them, the Thymidylate synthase gene (*TYMS*), located at band 18p11.32 has been studied [9]. Thymidylate synthase is a key enzyme in the folate metabolism originating from the diet, catalyzing the deoxyuridine monophosphate conversion for deoxythymidine monophosphate process of DNA synthesis. The conversion is essential for the production of thymidine, nucleotide required for DNA repair and synthesis [3].

In humans the *TYMS* gene is widely polymorphic, and variable number of tandem repetitions (VNRT) are found in the promoter enhancer region five primer - untranslated region (5’-UTR), in most cases, with two (2R) or three repetitions (3R) of a sequence of 28 bp [9]. Studies have shown that the homozygotes 3R/3R increase expression of *TYMS* mRNA when compared to cells in 2R/2R homozygotes [3,10,11]. In addition to these alleles, other rarer may have SNPs of 3R alleles, and other VNRTs [10,12,13].

Polymorphisms in *TYMS* gene may have effects on the stability of RNAm, and thus, affecting the translation level of the protein expression. These genotypic effects may contribute to individual susceptibility to BC [11,12], and have been related to pharmacogenetic variation in chemotherapy treatments [14,15].

Based on the few reports, our objective was to evaluate the frequency of polymorphisms of the promoter region enhancer 5’-UTR of the *TYMS* gene (2R and 3R), comparing the results between HBC and SBC patients. As the polymorphism acts in the cycle of folic acid, it may be involved in the etiology of both the second mutation hereditary cases, as in mutations in sporadic cases.

## Methods

The present study was developed in a total of 70 Caucasian individuals into two distinct groups (I) 33 patients with SBC (without family history), and (II) 37 patients with HBC (with family history), to from the metropolitan region of Campinas, which take the treatment at the Clinical Hospital of UNICAMP. The control group was compound by 134 healthy subjects (only females) without history of BC, ovarian or prostate cancer in the family [16], and a normal population without taking into account the BC history composed by 67 males and 67 females recruited in hospital hemocenter, to evaluated the Hardy-Weinberg equilibrium in a normal population.

The patients group was analyzed consecutively in our study. To be include in the HBC group the patient should have the following: (i) to have breast cancer below 35 years old and bilaterality; (ii) or, familiar with breast and/or ovarian cancer, one of whom diagnosed at the age below of 60 years old, or bilateral breast cancer; (iii) or, two or more cases of breast or ovarian cancer in the 1^st^ degree relatives, independently of presentation age of the neoplasia; (iv) or, two 1^st^ degree relatives with breast cancer, one diagnosed age below 45 years old; (v) or, two 1^st^ degree relatives with ovarian cancer; (vi) male relative with breast cancer, without taking age into account. To be including in the SBC group the patient should have the breast cancer, in other conditions as HBC (based on http://www.spsenologia.pt). The control group should have three characteristic: (i) no breast cancer diagnosed; (ii) no familiar historical to breast, ovarian or prostate cancer; (iii) the same age than case group (based on http://www.spsenologia.pt).

The patients and control group were matched by age. All patients and controls included were Caucasian woman to statistical analysis.

The study was approved by the Ethics Committee of the Medical Sciences Faculty of UNICAMP (CEP: N°913/2011, CAAE: 0812.0.146.000-11) and it was developed with biological material stored in the DNA Bank stored at the Laboratory of Molecular Genetics (Department of Medical Genetics, School of Medical Sciences - UNICAMP). All patients signed a consent form before beginning the study.

### Polymorphisms analysis of the promoter enhancer region 5’-UTR of the *TYMS* gene (2R and 3R)

The DNA was extracted by phenol-chloroform method from peripheral venous blood. The identification of VNRT polymorphisms of the 5’UTR region of the *TYMS* gene was performed by PCR following the protocol: 12 μL of sterile ultra-pure water; 4 μL of dNTP mix (1.25 mM each) (Life Technologies, Waltham, MA, United States of America); 0.3 μL of Taq DNA polymerase (5 U) (Life Technologies, Waltham, MA, United States of America); 2.5 μL of 10x PCR assay buffer (500 mM Tris pH9.0, 200 mM NH4SO4, 15 mM MgCl2); 4 μL of MgCl_2_ (50 mM); 1 μL of *primer* (20 pmol) (Biotech) *TYMSF* (*Forward*: 5’–GTGGCTCCTGCGTTTCCCCC–3’) and 1 μL of *TYMSR* (*Reverse*: 5’– CCAAGCTTGGCTCCGAGCCGGCCACAGGCATGGCGCGG–3’) [8]; resulting a final amount of 50 μL. The reaction was subjected to an initial denaturation step at 94.0°C for 5 minutes, followed by 35 cycles of denaturation at 94.0°C for 1 minute, annealing of primers (at 59.1°C for 1 minute, and extension at 72.0°C for 2 minutes, and 10 minutes of final incubation at 72.0°C. The PCR product analysis was developed in a 12% polyacrylamide gel electrophoresis.

### Statistical analysis

The statistical analyze between HBC and SBC groups was calculated using the Chi-square test Yates corrected and the Fisher Exact test through the software Statistical Package for Social Sciences v.17.0 (version 17, SPSS Inc., Chicago, IL) [17]. Associations between polymorphisms in the 5’-UTR region of *TYMS* and BC risk were obtained through the Odds Ratios (ORs) was calculated, considering a 95% confidence interval (CI) and p-values less to 0.05. *HardyWeinberg* imbalance has been evaluated for the three groups using the program *HAPLOVIEW*[18].

According to G*power 3.1.2 software, to a power of 80% in the analyses, we need, respectively, 52 and 56 subjects, to Fisher Exact test and Chi-square test.

## Results

A total of 204 subjects was included in this study, 70 with breast cancer (33 with SBC, and 37 with HBC), and 134 healthy subjects without family history of BC (Controls). Four (04) individuals who had rare alleles (3R/4R, in two individuals with SBC; and 2R/4R, in two control individuals) were excluded from statistical analyses. The SBC Group (p = 0.054) was Hardy-Weinberg (HW) equilibrium. However, a HW imbalance was found in the control group (p = 0.005) and HBC patients (p = 0.048). To normal group population, the sample was not in Hardy-Weinberg (X^2^ = 8.41, p < 0.005).

Polymorphisms frequencies found in tandem in the 5’UTR region (2R and 3R) of the *TYMS* gene are shown in Table 1. The 3R allele had a higher frequency than the 2R allele in individuals with cancer (SBC, HBC and BC) and controls. Heterozygotes genotype (2R/3R) frequencies were significantly higher in individuals with BC (in both, SBC and HBC groups) than in controls.

**Table 1:** **Frequency of genotypes and alleles of the *TYMS *gene in patients with breast cancer and control group**

**Groups**	**N**	**Genotypic Frequencies N (%)**			**Allelic frequencies N (%)**	
		**2R/2R**	**2R/3R**	**3R/3R**	**2R**	**3R**
**SBC**	31	4 (13)	22 (71)	5 (16)	30 (48)	32 (52)
**HBC**	37	3 (8)	25 (68)	9 (24)	31 (42)	43 (58)
**BC**	68	7 (10)	47 (69)	14 (21)	61 (45)	75 (55)
**Control**	132	36 (27)	49 (37)	47 (36)	121 (46)	143 (54)

N = Number of individuals used for analysis; n = (%) Frequencies; SBC = sporadic breast cancer; HBC = hereditary breast cancer; BC = breast cancer.

Data (Table 2) show no significant difference between allele frequencies (2R and 3R) found in patients with cancer (SBC and HBC) when compared with the controls. The low frequency of heterozygous genotype (2R/3R) in control individuals brought a bias, equating them to the results found for frequency of BC patients. However, when compared genotypic variations (2R/2R, 2R/3R and 3R/3R) of the TYMS gene between BC individuals and controls, there were statistical differences (Table 3).

**Table 2:** **Comparison of 2R and 3R alleles of the polymorphism in the *TYMS *gene among individuals with breast cancer and controls**

	**Allele 2R**	**Allele 3R**	**χ**^**2**^	***p***	**OR**	**CI (95%)**
**SBC**	30	32	0.05	0.82	1.11	(0.62-2.00)
**Control**	121	143				
**HBC**	31	43	0.22	0.64	0.64	(0.49-1.48)
**Control**	121	143				
**BC**	61	75	0.01	0.94	0.96	(0.62-1.49)
**Control**	121	143				

χ^2^ = Chi-square Test Yates Corrected; OR = Odds Ratio; CI = Confidence Interval; SBC = sporadic breast cancer; HBC = hereditary breast cancer; BC = breast cancer.

**Table 3:** **Comparison of genotypic variations of the *TYMS *among individuals with BC and controls**

	**Genotypic variant of the *TYMS***		
	**2R/2R**	**2R/3R**	**3R/3R**
**SBC**	04	22	05
**Control**	36	49	47
***χ***^***2***^	2.80	10.4	3.53
***p***	0.11	**0**.**001**	0.06
***OR***	0.30	4.14	0.35
**CI** (**95**%)	(0.13-1.21)	(**1**.**77**-**9**.**71**)	(0.12-0.97)
**HBC**	03	25	09
**Control**	36	49	47
***χ***^***2***^	5.94	9.68	1.9
***p***	**0**.**01**	**0**.**002**	0.27
***OR***	0.23	3.53	0.58
**CI** (**95**%)	(**0**.**06**-**0**.**81**)	(**1**.**63**-**7**.**65**)	(0.25-1.33)
**BC**	07	47	14
**Control**	36	49	47
***χ***^***2***^	2.46	17.13	0.39
***p***	0.12	<**0**.**001**	0.53
***OR***	0.45	3.79	0.47
**CI** (**95**%)	(0.19-1.11)	(**2**.**03**-**7**.**08**)	(0.24-1.52)

χ^2^ = Chi-square; OR = Odds Ratio; CI = Confidence Interval; In Bold = Significant Statistical Values; SBC = sporadic breast cancer; HBC = hereditary breast cancer; BC = breast cancer.

The genotypic comparison between SBC patients and the controls showed a significant association with 2R/3R genotype variants (OR = 4.14; CI95% = 1.77-9.71). Now, when compared individuals with HBC to the controls individuals, the association was present for the 2R/2R genotypes (OR = 0.23; CI95% = 0.06-0.81) and 2R/3R (OR = 3.53; CI95% = 1.63-7.65). In the comparison between BC patients (SBC plus HBC) and controls, the association was presented in the 2R/3R genotype in the BC group (OR = 3.79, IC95% = 2.03-1.11). An additional analyze was did considering the normal population versus BC, and we observed the same corresponding association. The 2R/3R patient had bigger OR to cancer when compared with other patients (OR = 3.764; CI95% = 2.02-7.13), considering the genotypes frequency to the normal population, 36, 49 and 47; respectively to 2R/2R, 2R/3R and 3R/3R genotypes.

## Discussion

Breast Cancer has been associated with polymorphisms in genes candidate to be disease modifiers, such as *CYP19* (Family 19 of the Cytochrome P450), *GSTP1* (Glutathione S-Transferase Protein), *TP53* (Tumor Protein 53), *P21* (Protein 21) [19], *MTHFR* (Methylenetetrahydrofolate Reductase) and *TYMS* (Thymidylate Synthase) [20] and, which are involved in events such as synthesis, methylation and DNA damage repair, and cellular activation in carcinogen metabolism and the metabolism of anticancer drugs. Gene variations, besides being related to risk factors for the development of BC, they can interfere with gene expression or activity level, and may be associated with different tumor phenotypes tumor [21-23]. The study of genetic variation may influence clinical management and better pharmacogenetic intervention to BC [24,25].

In the present study it was investigated whether polymorphisms in the *TYMS* gene, as a molecule with a key role in DNA synthesis, are associated with risk of development of BC as SBC and/or HBC. Although several studies have associated this gene with other cancers (colorectal, lung, pancreatic, gastric, and lymphoma) [26-32], this study is the first to correlate the HBC and SBC forms to polymorphisms in the 5’-UTR region of the *TYMS* gene in Brazilian population, in this way, is difficult to do association with others studies with similar characteristics.

Studies in other populations have found significative associations between the *TYMS* gene 5’-UTR variations and the BC development [2,11,12]. But the fact that the 2R/2R and 2R/3R variants (in HBC and BC/HBC/SBC, respectively), which would have a protective effect have presented relation, and risk effect, respectively, need to be better studied. What can be explained by the imbalance of *HardyWeinberg* found, especially in our control population or by other mechanisms that need to be better studied.

The 3R allele results in greater TYMS activity [33]. Therefore, the 2R/3R genotype should have intermediate activity. As the TYMS competes with MTHFR (Methylenetetrahydrofolate reductase) at the cycle of folic acid metabolism, the availability of 5,10-methylene THF (tetrahydrofolate), Trinh et al. (2002) [34], issued the hypothesis that the 3R variant could affect the levels of 5,10-methylene THF and this could lead to lower cell concentration of S-Adenosyl methionine and consequent decrease in DNA methylation. Thus, DNA hypomethylation, may increase susceptibility gene mutations or alter the expression of genes as protooncogenes or tumor suppressor, or would result in epigenetic changes that may initiate carcinogenesis. The association of *TYMS* polymorphism has been reinforced in a meta-analysis, being the 3R allele important in the BC risk [2].

The 3R/3R genotype for the *TYMS* gene has been associated with high levels of enzyme activity in tumors, and it have been considered to the best prognostic presented by chemosensibilizing (5-Flurouracil, 5-FU) to the BC. It shows the importance of genotypic characterization of the *TYMS 5*’-*UTR* of Brazilian population, suggesting that genotype of *TYMS* gene related to the number of repetitions in tandem can be, at least, partial indicator to the 5-FU chemotherapy [15,35-39]. The frequency found for triple homozygous (3R/3R), with worse prognosis, was around 16% to 24% in our population with BC, and 36% for the control population. The low number of individuals with the genotype 3R/3R in our population with the disease can be positive, due to chemotherapy efficiency than the other two genotypes (2R/2R and 2R/3R, in more than 75% of cases).

These observations emphasize the biological and clinical importance of the *TYMS* polymorphisms in relation to response and toxicity to chemotherapy [40], even more for being related to risk of BC found in this study. Thus, with pharmacogenomic studies it would be possible to understand the action of different drugs in combination with the chemotherapeutic effect and the association with the gene analyzed, and using polymorphisms in the *TYMS* gene as population biomarkers of severity to different types of cancer, especially the BC.

In our study, the Hardy-Weinberg in control group was not found because we selected only people without BC, and maybe as an important factor, the natural selection acts in the way to put better mechanisms in response to the cancer. In other study realized in our laboratory (data no divulgate) with an aneuploidia and controls we have the same data, the control population (woman) not shows Hardy-Weinberg equilibrium [63 woman; 23 (36.50%), 20 (31.75%), 20 (31.75%), respectively to 2R/2R, 2R/3R and 3R/3R genotypes frequency, p < 0.05]. As a major consideration, we can have a population to analyze the genotype frequency without taking into account a disease as selection factor, as we provide in the present study, as well we include only woman (only with adult advanced age) without BC our historical of the disease.

## Conclusion

We conclude that the prevalence of allelic polymorphisms 3R (*TYMS*) are increased when compared with the allele 2R in our population. The identification of alleles and genotypic variants of the promoter enhancer region *5*’-*UTR* of the *TYMS* (2R and 3R) showed that the differences found between patients with SBC and HBC are small. The bias found in the unbalance of Hardy-Weinberg into two groups (control and HBC) make impossible to say that there is an association between the *TYMS* and the development of breast carcinoma; but as the found results show an association, other studies would be necessary, increasing the number of individuals with lack of imbalance of constant allele frequencies found, for the same in the control group, in order to better understand the role in disease. But, in our data, an important aspect can be seen, as the important of 2R/3R genotypes in the BC risk in all groups analyzed, in this way, we need to have other studies, in different contexts to analyze this patients, not only by the genetic polymorphism, but including the protein analyze.

## Abbreviations

BC: Breast cancer; HBC: Hereditary breast cancer; BRCA1: Breast cancer 1 gene; BRCA2: Breast cancer 2 gene; SBC: Sporadic breast cancer; TYMS: Thymidylate synthase; DNA: Deoxyribonucleic acid; mRNA: Messenger ribonucleic acid; UTR: Untranslated region; Unicamp: University of campinas; dNTP: Deoxyribonucleoside triphosphates; CYP19: Cytochrome, family 19; P21: Protein 21; TP53: Tumor protein 53; GSTP1: Glutahione S-transferase P1; VNTR: Variable number in tandem repeats; SNPs: Single sequence polymorphism; MTHFR: Methylenetetrahydrofolate reductase; THF: Tetrahydrofolate.

## Competing interests

Authors declare that they have no competing interests.

## Authors’ contributions

JSNJ: Development of molecular analysis, writing of the manuscript, and literature review. FALM: data analysis and writing of the manuscript. CSB: writing of the manuscript, and responsibility for project. All authors read and approve the final manuscript.
